# Anti-Inflammatory Therapy for Temporomandibular Joint Osteoarthritis Using mRNA Medicine Encoding Interleukin-1 Receptor Antagonist

**DOI:** 10.3390/pharmaceutics14091785

**Published:** 2022-08-26

**Authors:** Jia Deng, Yuta Fukushima, Kosuke Nozaki, Hideyuki Nakanishi, Erica Yada, Yuki Terai, Kenji Fueki, Keiji Itaka

**Affiliations:** 1 Department of Biofunction Research, Institute of Biomaterials and Bioengineering, Tokyo Medical and Dental University (TMDU), Tokyo 101-0062, Japan; 2 Department of Masticatory Function and Health Science, Graduate School of Medical and Dental Sciences, Tokyo Medical and Dental University (TMDU), Tokyo 113-8549, Japan; 3 Department of Advanced Prosthodontics, Graduate School of Medical and Dental Sciences, Tokyo Medical and Dental University (TMDU), Tokyo 113-8549, Japan; 4 NanoCarrier Co., Ltd., Tokyo 104-0031, Japan; 5 Innovation Center of NanoMedicine (iCONM), Kawasaki Institute of Industrial Promotion, Kawasaki 210-0821, Japan

**Keywords:** osteoarthritis (OA), temporomandibular joint (TMJ), mRNA therapeutics, interleukin-1 receptor antagonist (IL-1Ra), polyplex nanomicelle

## Abstract

Messenger RNA (mRNA) is an emerging drug modality for protein replacement therapy. As mRNA efficiently provides protein expression in post-mitotic cells without the risk of insertional mutagenesis, direct delivery of mRNA can be applied, not only as an alternative to gene therapy, but also for various common diseases such as osteoarthritis (OA). In this study, using an mRNA-encoding interleukin-1 receptor antagonist (IL-1Ra), we attempted anti-inflammatory therapy in a rat model of the temporomandibular joint (TMJ) OA, which causes long-lasting joint pain with chronic inflammation. For the intra-articular injection of mRNA, a polyplex nanomicelle, our original polymer-based carrier, was used to offer the advantage of excellent tissue penetration with few immunogenic responses. While the protein expression was transient, a single administration of IL-1Ra mRNA provided sustained pain relief and an inhibitory effect on OA progression for 4 weeks. The mRNA-loaded nanomicelles provided the encoded protein diffusely in the disc and articular cartilage without upregulation of the expression levels of the pro-inflammatory cytokines IL-6 and tumor necrosis factor-α (TNF-α). This proof-of-concept study demonstrates how anti-inflammatory proteins delivered by mRNA delivery using a polyplex nanomicelle could act to alleviate OA, stimulating the development of mRNA therapeutics.

## 1. Introduction

Temporomandibular joint (TMJ) osteoarthritis (OA) is a degenerative joint disease characterized by the progressive degradation of cartilage, subchondral bone remodeling, and chronic pain [1,2]. The non-negligible pain and discomfort caused by TMJOA considerably reduces the quality of life of patients. The current clinical treatments are mainly palliative, such as analgesics and jaw exercises [3]. There is no available disease-modifying therapeutic strategy for arresting the progression of joint degeneration.

While the pathogenesis of TMJOA is considered multifactorial, inflammation is one of the most crucial factors that contribute to the progression of TMJOA [3]. Elevated expression levels of interleukin-1β (IL-1β) are found in the synovial fluid of TMJOA patients [4], and the expression of IL-1β in cartilage and synovial tissue is closely associated with OA severity and arthralgia [5,6]. The binding of IL-1 to IL-1 receptor 1 (IL-1R1) triggers downstream-signaling pathways, which further stimulate other inflammatory cytokines, such as IL-6, and induce the synthesis of matrix metalloproteinases [7,8,9]. From the perspective of developing disease-modifying OA drugs, one of the most efficient candidates is the IL-1 receptor antagonist (IL-1Ra). IL-1Ra binds to IL-1R1, but is unable to activate the IL-1 intracellular signaling pathways [10]. Based on this property, the recombinant IL-1Ra was demonstrated to ameliorate OA progression in animal models [11,12], and clinically used as an agent named anakinra in various anti-inflammatory therapies since its approval by the FDA [13,14,15,16]. An early and significant suppression of joint pain by a single injection of anakinra was observed in a clinical trial, but the effect was not sustained [14].

Nucleic-acid-based drugs have gained considerable attention in recent years. While DNA therapies allow for the continuous production of the protein of interest, there are safety concerns, such as the insertion into the host genome, in addition to the problems of relatively low transfection efficiency [17,18]. In contrast, direct delivery of messenger RNA (mRNA) into cells has recently come into focus as a revolutionary technology for protein replacement therapy [19]. It efficiently achieves transient protein expression once it enters the cytoplasm, allowing it to be functional in post-mitotic cells and avoiding the risk of insertional mutagenesis [20,21]. Thus, in addition to being an alternative to gene therapy, mRNA can be widely used for various common diseases such as OA. Lipid nanoparticles (LNP) are the most commonly used mRNA delivery methods, but the immune–stimulation property of the LNP may interfere with the therapeutic effect of the mRNA, especially for anti-inflammatory therapy [22,23]. We developed an mRNA delivery system, a polyplex nanomicelle, based on the self-assembly of mRNA and a block copolymer comprising a polyethylene glycol (PEG)-polyamino acid (Poly{N-[N′-(2-aminoethyl)-2-aminoethyl]aspartamide}) block copolymer (PEG-PAsp(DET)) [24,25,26]. Other than the mRNA-loading capability, the nanomicelle has little or no effect on immune stimulation because of the surface covered by a dense PEG palisade [27]. Using these nanomicelles, mRNA could be administered to target cells with negligible immune reactions, even when using wild-type mRNAs [28,29,30,31,32,33,34,35,36] (see Table S1) [22,23,24,25,26,27,28,29,30,31,32,33,34,35,36,37,38,39,40,41,42,43,44].

In this study, we conducted an anti-inflammatory therapy for TMJOA using mRNA-loaded polyplex nanomicelles. IL-1Ra mRNA was introduced into the TMJ, followed by the evaluation of its therapeutic effects, including pain relief and prevention of tissue degradation, demonstrating the feasibility of nanomicelles for the treatment of articular inflammation using mRNA medicine.

## 2. Materials and Methods

### 2.1. Chemicals

The β-benzyl-L-aspartate N-carboxyanhydride (BLA-NCA) was purchased from Chuo Kasei Co., Ltd. (Osaka, Japan). The α -methoxy-ω-amino poly(ethylene glycol) (PEG-NH2) (Mw 42k) was obtained from Nippon Oil and Fats (Tokyo, Japan); diethylenetriamine (DET) and 2-[4-(2-hydroxyethyl)-1-piperazinyl]ethanesulfonic acid (HEPES) were purchased from Wako Pure Chemical Industries, Ltd. (Osaka, Japan). DET was used after conventional distillation.

### 2.2. Synthesis of Block Copolymers

The block copolymers were synthesized as previously described [24]. Briefly, the polymerization of β-benzyl-L-aspartate N-carboxyanhydride (BLA-NCA) (Chuo Kasei, Osaka, Japan) was initiated from the terminal primary amino group of PEG-NH_2_ to obtain PEG-b-PBLA. Subsequently, diethylenetriamine (DET) was introduced into the side chain of PBLA via an amino lysis reaction. Gel permeation chromatography measurements confirmed that the synthesized block polymers had a narrow unimodal molecular weight distribution (Mw/Mn = 1.04). The polymerization degree of the DET segment was determined to be 63 using ^1^H NMR spectroscopy (JEOL EX300 spectrometer, JEOL, Tokyo, Japan).

### 2.3. Preparation of mRNA

Untagged human IL-1Ra open reading frame (ORF) sequences were purchased from Thermo Fisher Scientific (Waltham, MA, USA). To construct a DNA template for in vitro transcription, the coding region of human IL-1Ra was cloned into the pSP73 vector (Promega, Madison, WI, USA) for expression under the T7 promoter. A 120-bp poly A/T sequence was cloned into the vector downstream of the protein-coding sequence to attach a poly (A) chain to the mRNA 3′ terminal. In vitro transcription was performed on linearized pSP73-IL-1Ra-Poly(A) using the mMESSAGE mMACHINE T7 Ultra Kit (Ambion, Carlsbad, CA, USA), followed by RNA purification using the RNeasy Mini Kit (Qiagen, Hilden, Germany), according to the manufacturer’s instructions. Unmodified ribonucleic acid triphosphates were used for in vitro transcription. The quantity and quality of the transcribed mRNA were determined using a Nanodrop 2000 spectrophotometer (Thermo Fisher Scientific, Waltham, MA, USA) and an Agilent 2100 Bioanalyzer chip-based capillary electrophoresis system (Agilent Technologies, Santa Clara, CA, USA), respectively. Finally, mRNAs encoding with luciferase 2 (Luc2) (Promega) or green fluorescent protein (ZsGreen1) (pZsGreen1-N1; Takara Bio Inc., Shiga, Japan) were prepared as described above.

### 2.4. Preparation of mRNA-Loaded Polyplex Nanomicelles

Polyplex nanomicelles were prepared by mixing solutions of mRNA, a block copolymer (PEG-PAsp (DET)), and 10 mM of HEPES buffer (pH 7.3). The concentration of mRNA was set at 75 μg/mL, and that of PEG-PAsp (DET) was adjusted for obtaining an N/P ratio (the residual molar ratio of the polycations amino groups to the mRNA phosphate groups) to be 8. The final mRNA concentration was 50 μg/mL.

### 2.5. Animal Model

All animal experimental procedures were approved by the Institutional Animal Care and Use Committee of the Tokyo Medical and Dental University (protocol number: A2020-174C4). Male SD rats (8 weeks old, with a mean weight of 240 g; Sankyo Labo, Japan) were used in this study. Rats were housed at room temperature with a 12 h light/12 h dark cycle and allowed access to food and water ad libitum. TMJOA was induced by intra-articular injection of monosodium iodoacetate (MIA) (Sigma-Aldrich, St. Louis, MO, USA), as previously described [37]. Briefly, under 2% isoflurane anesthesia and 0.5 mg of MIA dissolved in 50 μL of PBS was injected using a 30-gauge needle into the upper compartment of the bilateral joints. One day after the MIA injection, 50 μL of nanomicelle solution containing 2.5 μg of mRNA was injected into the TMJs for further evaluation.

### 2.6. Pain Behavior Assessment

Mechanical nociception was assessed based on the head withdrawal threshold (HWT) using von Frey microfilaments (Muromachi Kikai Co., Tokyo, Japan) [38]. The TMJ areas of rats were tested from the lowest force of the filaments to determine HWT, which was defined as the lowest force to produce a reflex response. The tests were performed at least three times for each rat. The HWT was calculated as the mean value per joint of six rats/group (in total, 12 joints/group).

### 2.7. Micro-Computed Tomography Evaluation

Two and four weeks after mRNA injection, the animals were euthanized to dissect the bilateral TMJs. The harvested TMJ condyles were fixed overnight in 4% paraformaldehyde and analyzed using a high-resolution micro-computed tomography (micro-CT) system (inspeXio SMX-100CT; SHIMADZU, Kyoto, Japan). The samples were scanned at 90 kV and 65 μA, with an effective pixel size of 8 μm. Sagittal condylar images were reconstructed.

### 2.8. Histological and Immunofluorescence Analyses

After the micro-CT examination, the fixed TMJ specimens were dehydrated in 20% sucrose solution and embedded in carboxymethyl cellulose for frozen sections using Kawamoto’s film method [39]. Serial sections were cut in the sagittal direction at 3-μm and stained with toluidine blue (TB) and safranin-O (SO) for cartilage evaluation according to the standard protocols. Based on TB- and SO-stained sections, cartilage degradation and repair were evaluated by three blind independent observers using Mankin’s scoring system [40,41]. The TB-stained sections were also used to measure the thickness of cartilage in each region using ImageJ software version 1.53 (National Institutes of Health, Bethesda, MD). For immunofluorescence staining, sections were incubated with antibodies against ZsGreen1 (1:250, rabbit polyclonal, Takara Bio USA, San Jose, CA, USA) and Alexa Fluor-488 goat anti-rabbit secondary antibodies (1:250, Invitrogen, Carlsbad, CA, USA) according to standard protocols. The sections were then counterstained with DAPI (Thermo Fisher Scientific, Waltham, MA, USA) and observed under an inverted fluorescence microscope (BZ9000; Keyence Co., Itasca, IL, USA).

### 2.9. Quantitative Reverse Transcription Polymerase Chain Reaction (qRT-PCR)

The total RNA was isolated from only the discs and cartilage of the condylar heads using the RNeasy Fibrous Tissue Kit (Qiagen, Hilden, Germany) according to the manufacturer’s instructions as recommended by a previous report [42]. Bilateral condyle heads of each rat were counted as one sample for extracting sufficient RNA. The tissue was frozen in liquid nitrogen immediately after dissection and homogenized using a Multi-Beads Shocker (Yasui-kikai, Osaka, Japan). Reverse transcription was performed using the PrimeScript RT Master Mix (Takara Bio, Shiga, Japan). The qRT-PCR was performed with a PowerTrack™ SYBR™ Green Master Mix (Applied Biosystems, Foster City, CA, USA) using the StepOnePlus™ Real-time PCR system (Applied Biosystems). Amplification specificity was confirmed using melting curves. Relative mRNA expression levels were normalized to that of the housekeeping gene β-actin and calculated using the ΔΔCT method. The sequences of the gene-specific primers are listed in Table 1.

### 2.10. Western Blot

The bilateral condylar heads of each rat were counted as one sample to extract sufficient protein. The total protein was collected from the discs and cartilages of the condylar heads. The tissue was frozen immediately after dissection and then homogenized using a Multi-Beads Shocker (Yasui-kikai, Osaka, Japan) after adding a mixture of RIPA buffer and Pierce™ Protease Inhibitor Tablets (Thermo Fisher Scientific, Waltham, MA, USA). The total protein concentration was determined using the Pierce™ BCA protein assay kit (Thermo Fisher Scientific, Waltham, MA, USA), and 40 μg of protein samples were subjected to sodium dodecyl sulfate-polyacrylamide gel electrophoresis using an MES SDS running buffer (Thermo Fisher Scientific, Waltham, MA, USA). Subsequently, blots were transferred onto polyvinylidene fluoride membranes using the Trans-Blot^®^ Turbo™ Transfer System (BIO-RAD Laboratories, Hercules, CA, USA). The membrane was then blocked in a blocking buffer (5% non-fat milk in Tris-buffered saline containing 0.05% Tween-20 (TBST)) at room temperature for 1 h, and incubated overnight at 4 °C with primary antibodies against either the anti-human IL-1Ra antibody (1:100, mouse monoclonal Santa Cruz Biotech, Dallas, TX, USA) or anti-GAPDH (1:5000, mouse monoclonal, Sigma-Aldrich, St. Louis, MO, USA). After washing in TBST three times (10 min per wash), the membrane was incubated with an HRP-conjugated goat anti-mouse secondary antibody (Promega, Madison, WI, USA). The membrane was washed in TBST three times (10 min per wash), followed by incubation in SuperSignal™ West Dura Extended Duration Substrate (Thermo Fisher Scientific, Waltham, MA, USA) for enhanced chemiluminescence. Finally, the blots were exposed for 2 min in an iBright™ CL1500 Imaging System (Thermo Fisher Scientific, Waltham, MA, USA) for detection.

### 2.11. Statistical Analysis

All pooled data for the qRT-PCR, pain behavior tests, and histological analyses are presented as mean ± standard error of the mean (SEM). Differences between groups were analyzed using GraphPad Prism 8 (GraphPad Software, San Diego, CA, USA) using a two-way ANOVA, followed by Tukey’s multiple comparison test. Statistical significance was set at *p* < 0.05.

## 3. Results

### 3.1. Evaluation of mRNA Delivery into the Articular Cartilage

**Figure 1:**
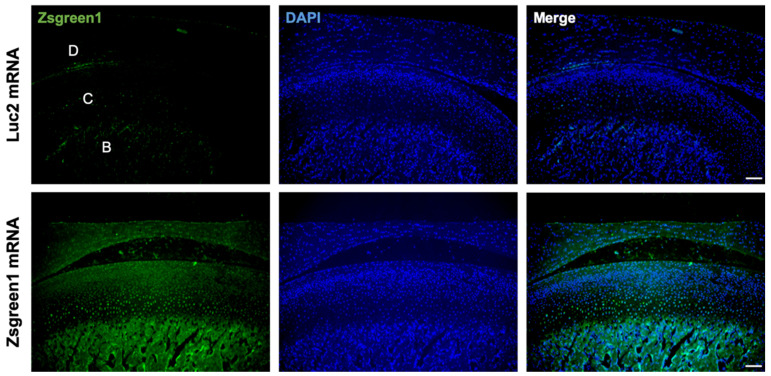
Diffusive ZsGreen1 protein expression from the exogenous mRNA in the temporomandibular joint (TMJ). Representative images after immunofluorescence staining by anti-ZsGreen1 antibody (Green) and DAPI (Blue). D, disc. C, cartilage. B, bone. Scale bar, 100 μm.

**Figure 2:**
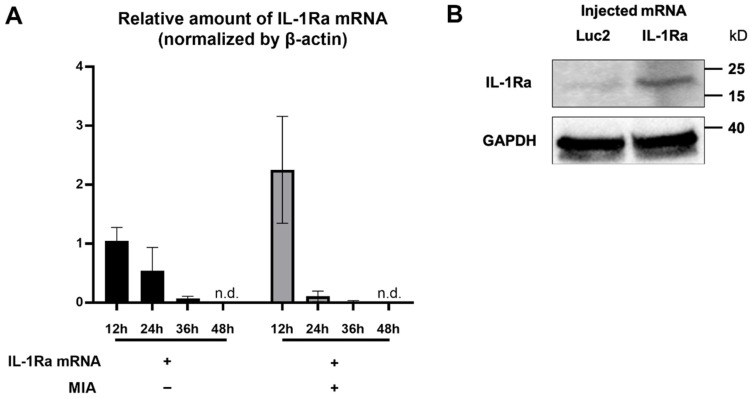
Transient interleukin-1 receptor antagonist (IL-1Ra) protein production from the exogenous mRNA delivered into the articular cartilage. (**A**) Relative amount of the exogenous IL-1Ra mRNA detected in the cartilage tissue by qRT-PCR at 12, 24, 36, and 48 h after the mRNA injection. *n* = 3/group. Data are presented as mean ± SEM. n.d., not detected. (**B**) Protein production of IL-1Ra from the exogenous mRNA at 24 h post mRNA injection, evaluated by Western blotting. Luciferase 2 (Luc2) mRNA was used as a negative control.

To investigate the feasibility of mRNA delivery for intra-articular treatment, ZsGreen1 mRNA was injected into the TMJs, and the distribution of protein expression was visualized by immunofluorescence staining 24 h after the injection. The signals of ZsGreen1 were observed diffusively in the disc and articular cartilage for the ZsGreen1 mRNA-injected group, whereas almost no signals were detected for the negative control group receiving luciferase (Luc2) mRNA (Figure 1). To further evaluate the delivery of IL-1Ra mRNA into the cartilage, as well as the expression of the IL-1Ra protein, only cartilaginous tissue including the disc was collected after mRNA injection, and the relative amount of IL-1Ra mRNA was measured by a qRT-PCR. As shown in Figure 2A, the relative amount of IL-1Ra mRNA in the cartilage was the highest 12 h after mRNA injection. While the relative amount of IL-1Ra mRNA gradually decreased, it remained detectable in chondrocytes at 36 h post-injection. The relative amount of IL-1Ra mRNA reached a level undetectable by a qRT-PCR 48 h after injection. To further evaluate the delivery of mRNA nanomicelles under the inflammatory status, an MIA-induced TMJOA model was used, in which MIA injected into the joint cavity induced acute inflammation, resulting in continuous pain as well as progressive cartilage degeneration [37]. Figure 2A shows that the trend of the relative amount of exogenous mRNA detected in the cartilaginous tissue was not altered by MIA injection, indicating that the mRNA nanomicelles were similarly delivered under the inflammatory condition. IL-1Ra expression was also confirmed at the protein level at 24 h post-injection by Western blotting and compared with that of the negative control Luc2 mRNA-injected group (Figure 2B). Taken together, IL-1Ra mRNA was capable of transient protein production after delivery into the TMJ cartilage.

### 3.2. IL-1Ra mRNA Continuously Suppresses Pain from an Early Time Point

**Figure 3:**
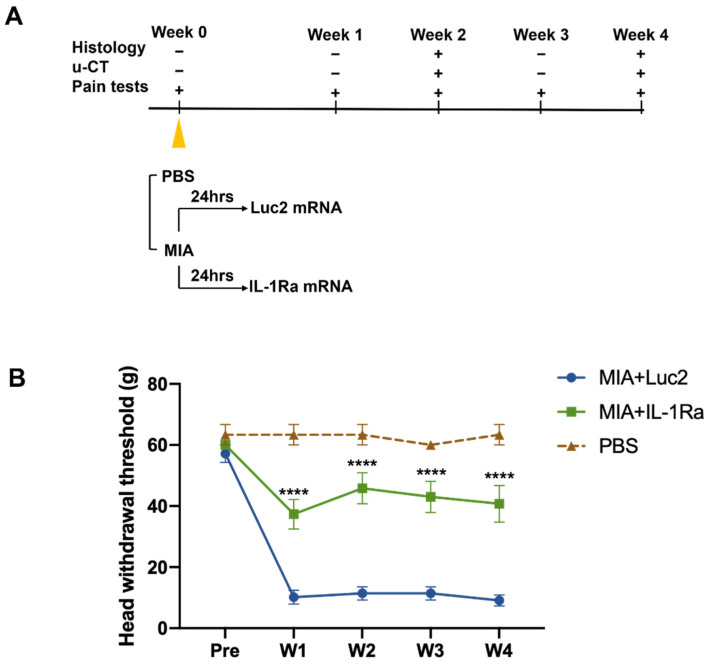
Pain behaviors before and after the intra-articular injection of the interleukin-1 receptor antagonist (IL-1Ra) mRNA. (**A**) Schematic outline of the treatment design. Monosodium iodoacetate (MIA, 0.5 mg) dissolved in 50 μL PBS was injected into the upper compartment of the temporomandibular joint (TMJ) to induce temporomandibular joint osteoarthritis (TMJOA). After 24 h, the rats received an intra-articular injection of 2.5 μg IL-1Ra mRNA in 50 μL using nanomicelles. Injection of luciferase 2 (Luc2) mRNA served as the untreated control. Pain behaviors were monitored weekly. TMJ samples were harvested at 2 and 4 weeks post the mRNA injection for histological analysis and micro-CT. (**B**) Pain behaviors evaluated by head withdrawal threshold (HWT). Pain was significantly reduced by IL-1Ra mRNA treatment from the first week. Animal number = 6/group. Data are presented as mean ± SEM. **** *p* < 0.0001, comparison between the IL-1Ra and Luc2 groups using two-way ANOVA followed by Tukey’s multiple comparison test.

The MIA-induced TMJOA model is an established method for investigating the mechanism of OA-like pain and its treatment [43,44,45]. To treat TMJOA using mRNA therapeutics, we injected IL-1Ra mRNA intra-articularly into the bilateral TMJs at 1 d after the MIA injection (Figure 3A). To investigate the effects of a single injection of IL-1Ra mRNA on pain behaviors, nociceptive responses were assessed by the HWT weekly until the fourth week after treatment with IL-1Ra mRNA (Figure 3B). Surprisingly, after the MIA injection, rats injected with Luc2 mRNA showed a significantly decreased head withdrawal threshold (HWT) from the first week, but there was no improvement throughout the experiments. In contrast, rats treated with IL-1Ra mRNA had significantly reduced pain behaviors compared with those treated with the negative control (Luc2) mRNA for 4 weeks after a single administration of the mRNA, as indicated by the significantly higher HWT than those of the Luc2 mRNA-injected rats as early as 1 week post-treatment, and this effect was maintained until the end of monitoring.

### 3.3. IL-1Ra mRNA Alleviates Degeneration of Cartilage and Bone

**Figure 4:**
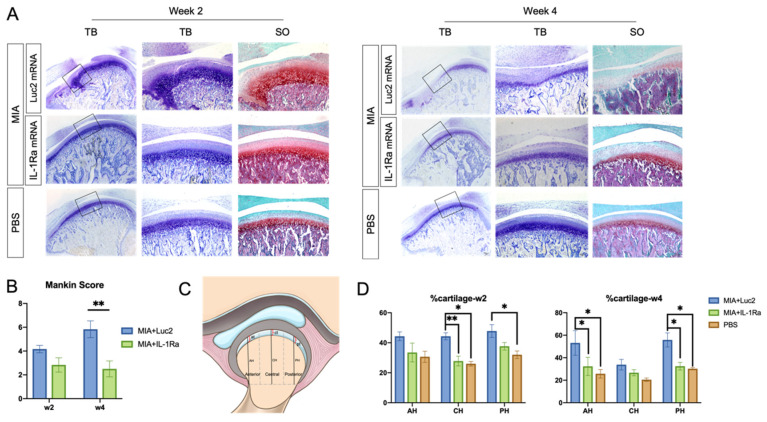
Histological analysis of rat temporomandibular joint osteoarthritis (TMJOA) cartilage followed by interleukin-1 receptor antagonist (IL-1Ra) mRNA administration. (**A**) Images of representative sections stained with hematoxylin and eosin (HE), toluidine blue (TB), and safranin-O (SO) at 2 and 4 weeks after the injection of 2.5 μg IL-1Ra or luciferase 2 (Luc2) mRNAs using polyplex nanomicelles, into the monosodium iodoacetate (MIA)-treated temporomandibular joints (TMJs). Joint number = 6/group. (**B**) Mankin scores of MIA-treated TMJs 2 or 4 weeks after injection of IL-1Ra or Luc2 mRNAs. Joint number = 6/group. (**C**) Schematic illustration of the measurements in rat TMJ condylar heads. AH: anterior height; CH: central height; PH: posterior height of condyle; at: anterior thickness; ct: central thickness; and pt: posterior thickness of cartilage. Cartilage thickness was measured and expressed as percentage of condylar height at different regions (% at, % ct, and % pt). (**D**) Cartilage thickness at 2 and 4 weeks. Joint number = 6/group. Data represent mean ± SEM. * *p* < 0.05, ** *p* < 0.01, analyzed by two-way ANOVA followed by Tukey’s multiple comparison test.

To evaluate the effect of a single administration of IL-1Ra mRNA on the cartilage and bone, we did histologic analyses 2 and 4 weeks after the mRNA injection. At 2 weeks after mRNA injection, the Luc2 mRNA-injected group showed a loss of chondrocytes in the anterior areas of the condyles, disruption of the osteochondral junction in the central region, and chondrocyte proliferation with deep matrix staining in the central and posterior regions (Figure 4A), which was consistent with a previous report using the MIA-induced TMJOA model [37]. In contrast, compared to the Luc2 group, IL-1Ra mRNA decreased the changes in the cartilage and bone–cartilage junction, shown as regularly arranged layers of chondrocytes and less chondrocyte proliferation in the central area (Figure 4A). While the Mankin score of the IL-1Ra mRNA group was not significantly lower than that of the Luc2 group (Figure 4B), the percentage of cartilage, which was reported to indicate fibrous surface thickening (Figure 4C) [43], was almost maintained at the level of normal TMJ (age-matched PBS group) in the IL-1Ra-treated group, whereas the Luc2-injected group showed an increase in fibrous surface thickening compared with the PBS- and IL-1Ra mRNA-treated groups (Figure 4D).

**Figure 5:**
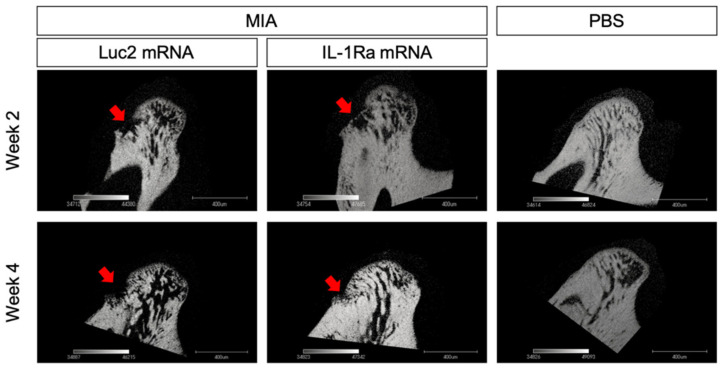
Radiographic changes in the condylar subchondral bone. Micro-CT images of the sagittal view of the condyles of monosodium iodoacetate (MIA)-treated temporomandibular joints (TMJs) with receiving luciferase 2 (Luc2) or interleukin-1 receptor antagonist (IL-1Ra) mRNAs, as well as PBS-treated TMJ. Arrows indicate the bone erosion on the surface of the condyle.

At 4 weeks post the mRNA injection, the differences between the IL-1Ra mRNA and Luc2 mRNA groups became highly conspicuous. The Luc2-injected group exhibited typical OA progression, including cartilage destruction with severe loss of staining, chondrocyte clustering, subchondral bone erosion, and osteophyte formation (Figure 4A). In contrast, IL-1Ra mRNA ameliorated these changes, which was clearly demonstrated by the significantly lower Mankin scores than those of the Luc2-injected group (Figure 4B). Although mild surface fibrosis was observed in the IL-1Ra mRNA-treated group compared with that in the PBS group (Figure 4A,D), the morphological changes, such as peripheral cartilage thickening in the posterior area, were much less than those in the Luc2 group (Figure 4D). In addition, the effect of reducing OA progression by IL-1Ra mRNA, especially in the bony structure, was observed in the micro-CT images of the condyle (Figure 5). While bony erosions appeared on the surface of the condyle in the MIA-injected groups, the size of the bone defect was smaller in the IL-1Ra mRNA group than in the Luc2 mRNA group, strongly suggesting alleviation of joint inflammation by a single administration of IL-1Ra mRNA.

### 3.4. IL-1Ra mRNA Modulates OA-Induced Inflammation

**Figure 6:**
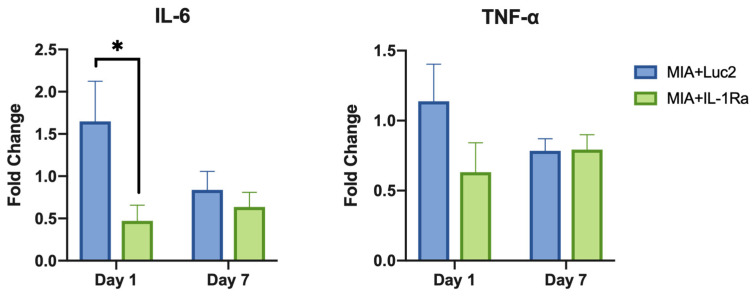
Suppression of inflammatory cytokines after intra-articular delivery of interleukin-1 receptor antagonist (IL-1Ra) mRNA. The expression levels of pro-inflammatory cytokines, including IL-6, and tumor necrosis factor (TNF)-α at 1 d and 7 d after the mRNA treatment. Fold change shows the relative expression compared with the expression level in healthy joints. β-actin expression was used for normalization. The expression levels of IL-6 were significantly lower in the IL-1Ra group than those in the luciferase 2 (Luc2) group on Day 1. Animal number = 6/group. * *p* < 0.05, analyzed by two-way ANOVA followed by Tukey’s multiple comparison test.

Finally, to investigate the immune-response modulation of IL-1Ra mRNA therapy, gene expression of pro-inflammatory cytokines in the cells of MIA+Luc2, MIA+IL-1Ra, and healthy TMJ cartilage was evaluated by qRT-PCR on days 1 and 7 after the mRNA administration. While the expression level of IL-1β was not altered by the IL-1Ra mRNA (Figure S1), the downstream-signaling cytokines, such as IL-6 and TNF-α, showed remarkable downregulation (Figure 6). In the MIA+Luc2 group, the expression level of the pro-inflammatory cytokine IL-6 was approximately 1.5 times that of healthy joints 1 d after the Luc2 mRNA injection, but the expression of IL-6 was significantly downregulated in the IL-1Ra group compared to that in the Luc2 group on day 1 after the mRNA injection. However, on day 7, the levels of IL-6 expression decreased in parallel in both the IL-1Ra and Luc2 groups. TNF-α levels showed similar changes after the mRNA injection, although there were no significant differences between the two groups. These results are compatible with the potential role of IL-1Ra mRNA in suppressing cartilage inflammation by inhibiting the downstream cytokines of IL-1.

## 4. Discussion

In this study, we demonstrated the therapeutic effects of IL-1Ra mRNA on TMJOA. By alleviating joint inflammation, the mRNA exhibited therapeutic effects in reducing pain and suppressing OA progression in the cartilage and subchondral bone.

To achieve the anti-inflammatory effect, the polyplex nanomicelle played a critical role. The uniform distribution of expression in the target cartilage and subchondral bone (Figure 1) is one of the most characteristic features of nanomicelles. This is because of the nanomicelle properties of having a well-regulated particle size of several tens of nanometers, with the surface covered by a dense PEG palisade, providing excellent tissue permeability [27]. In addition, the less or non-immunogenic behavior of the nanomicelles should also be an important factor in achieving a therapeutic effect. Currently, LNPs are mostly used as mRNA carriers, especially for mRNA vaccines [46,47,48]. While LNP has a high capacity for delivering mRNA, LNP potentially elicits innate immune pathways (Table S1) [49], which may improve the performance of vaccination, but be undesirable for therapeutic agents. Although the immune responses after the injection of nanomicelles into the normal joint (not TMJOA) were not evaluated in this study, the nanomicelles have been confirmed to have the capacity to administer mRNA with hardly any immune responses in our previous studies on injecting mRNA into the knee joint [30], intervertebral disc [33], spinal cord [32], and brain [34,36]; the dense PEG surface of the nanomicelles contributes to minimal interaction with the plasma membrane. The sophisticated polymer design of a pH-responsive character allows the smooth endosomal escape of the mRNA-loaded nanomicelles, resulting in circumventing the recognition of the mRNA by TLRs [26,28]. Of note, the mRNA used in this study was of a wild type, not containing modified nucleotides such as pseudouridine to reduce the immunogenicity [50]. While the modified mRNA may preferably be used in clinical practice, the experimental results of joint inflammation suppression, even using wild-type mRNA obtained in this study, demonstrate the usefulness of the nanomicelles for mRNA delivery without inducing immune responses.

The noteworthy outcome in this study was that a single intra-articular injection of IL-1Ra mRNA was capable of suppressing pain behaviors for one month. This is partly because, unlike anakinra, which has a short half-life of only 4 h [14], the mRNA provided the protein for more than one day (Figure 2A). The duration of the protein expression was apparently quite short compared with that of pain relief. However, the pathogenic mechanism of chronic pain may be related to sustained therapeutic effects. In OA, chronic joint pain is likely to be triggered by altered neuronal activity, including the sensitization of the peripheral primary sensory neurons and central nociceptive neurons [51,52,53,54]. Sensitization of nociceptive neurons is generated and maintained by inflammation within the peripheral or central neuronal system, and leads to chronic pain [55,56]. In the TMJOA model used in this study, MIA induced inflammation in the joint with the upregulation of inflammatory cytokines, including IL-6 and TNF-α, followed by cartilage degeneration and bone destruction for several weeks. It was demonstrated in a previous study that, the hyperalgesia of TMJ in the first week after MIA induction could mainly be attributed to an inflammatory response [37]. Our findings show that IL-1Ra mRNA treatment significantly reduced the expression level of IL-6 as early as 24 h post treatment compared to the Luc2 control group (Figure 6), indicating that IL-1Ra, as the natural inhibitor of the IL-1 signaling pathway, suppressed TMJOA pain by blocking the MIA-induced inflammatory cascade at an early time point. Moreover, the time course of inflammation induced by MIA itself tends to be rapid, lasting only for a few days [57]. Our results evaluating the expression of inflammatory cytokines (Figure 6), where the expression spontaneously decreased on day 8 after MIA introduction (day 7 after the mRNA treatment), even for the negative control group receiving Luc2 mRNA, is consistent with the time course of MIA-induced inflammation. This aspect of MIA-induced OA may be closely related to the sustained effect of a single administration of IL-1Ra mRNA to prevent OA progression by alleviating acute inflammation.

There are some limitations in this study. First, the OA model induced by MIA is represented by acute inflammation, not directly mimicking the complex conditions of chronic OA. Furthermore, there is still a lack of a desirable model that possesses all the clinical features [3]. In addition, the mechanism of action of IL-1Ra, including the effect on inflammatory signaling, such as extracellular matrix molecules, metalloproteinases, and inflammatory cytokines, which play key roles in OA progression [58], still needs to be clarified in the future. Nevertheless, the results of this study open new possibilities for mRNA medicine for the use of known proteins for anti-inflammatory therapy. The beneficial effects would not necessarily be identical to those of protein administration. The nanomicelle would be an important option for less or non-immunogenic mRNA delivery. We believe that this mRNA medicine-based approach would offer many new options for the use of various therapeutic proteins.

## 5. Conclusions

This study demonstrates the feasibility of IL-1Ra mRNA for the treatment of TMJOA. A single administration of the mRNA using a polyplex nanomicelle provided sustained pain relief and an inhibitory effect on OA progression for 4 weeks. The nanomicelles provided the encoded protein diffusely in the disc and articular cartilage without upregulating the expression levels of the pro-inflammatory cytokines IL-6 and TNF-α. This proof-of-concept study demonstrates how anti-inflammatory proteins delivered by mRNA delivery using a polyplex nanomicelle could act to alleviate OA, stimulating the development of mRNA therapeutics.

## Figures and Tables

**Table 1:** Primers used for qRT-PCR.

Gene	Forward	Reverse
**Human**		
IL-1Ra	5′-AACCTTCTACCTGCGGAACA-3′	5′-GCCAGACTTCACACAGCTCA-3′
**Rat**		
β-Actin	5′-CACCCGCGAGTACAACCTTCT-3′	5′-TCGTCATCCATGGCGAACTGG-3′
IL-1β	5′-TGTCTGACCCATGTGAGCTG-3′	5′-TTTGGGATCCACACTCTCCA -3′
IL-6	5′-GTTTCTCTCCGCAAGAGACTTC -3′	5′-TGTGGGTGGTATCCTCTGTGA-3′
TNF-α	5′-ACTGAACTTCGGGGTGATCG-3′	5′-TCCGCTTGGTGGTTTGCTAC-3′

## Supplementary Materials

The following supporting information can be downloaded at: https://www.mdpi.com/article/10.3390/pharmaceutics14091785/s1, Figure S1: The expression levels of Interleukin-1β (IL-1β) on the 1st day and 7th day after the mRNA treatment. β-actin expression was used for normalization. Animal number = 6/group. Table S1. Comparison of physicochemical properties between lipid nanoparticles and polyplex nanomicelles for loading mRNA. References [22,23,24,25,26,27,28,29,30,31,32,33,34,35,36,46,59,60,61,62,63,64,65] are cited in the Supplementary Materials.
