# Supplementary material for: Anti-Inflammatory Therapy for Temporomandibular Joint Osteoarthritis Using mRNA Medicine Encoding Interleukin-1 Receptor Antagonist
Source: Pharmaceutics. 2022 Aug 26;14(9):1785. doi: 10.3390/pharmaceutics14091785 (PMC9505648; doi:10.3390/pharmaceutics14091785)
Supplement: Supplementary file 1 [file pharmaceutics-14-01785-s001.zip › pharmaceutics-1844241-supplementary.pdf]

Supplemental Materials

Anti-inflammatory Therapy for Temporomandibular Joint Osteoarthritis Using mRNA Medicine Encoding Interleukin-1 Receptor Antagonist

Jia Deng[1,2], Yuta Fukushima[1], Kosuke Nozaki[3], Hideyuki Nakanishi[1], Erica Yada[1,4], Yuki Terai[1], Kenji Fueki[2], Keiji Itaka[1,5*]

[1]Department of Biofunction Research, Institute of Biomaterials and Bioengineering, Tokyo Medical and Dental University (TMDU), 101-0062 Tokyo, Japan;
[2] Department of Masticatory Function and Health Science, Graduate School of Medical and Dental Sciences, Tokyo Medical and Dental University (TMDU), 113-8549 Tokyo, Japan;
[3] Department of Advanced Prosthodontics, Graduate School of Medical and Dental Sciences, Tokyo Medical and Dental University (TMDU), 113-8549 Tokyo, Japan;
[4]NanoCarrier Co., Ltd. 104-0031 Tokyo, Japan
[5]Innovation Center of NanoMedicine (iCONM), Kawasaki Institute of Industrial Promotion, 210-0821 Kawasaki, Kanagawa, Japan

Supplemental Figure S1

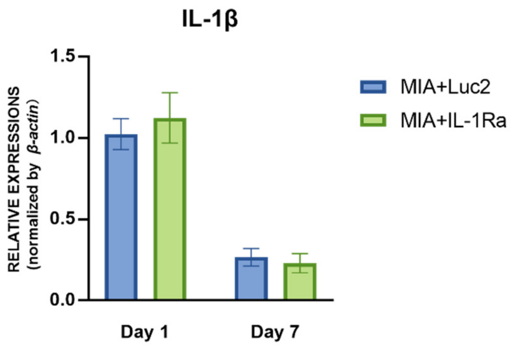

Figure S1. The expression levels of Interleukin-1β (IL-1β) on the 1st day and 7th day after the mRNA treatment. β-actin expression was used for normalization. Animal number = 6/group.

Table S1. Comparison of physicochemical properties between lipid nanoparticles and polyplex nanomicelles for loading mRNA.

	Lipid Nanoparticles-mRNA	Polyplex Nanomicelles
**Components**	Cationic/ionizable lipids and helper lipids such as phospholipids, cholesterol, or polyethylene glycol (PEG)-functionalized lipids (PEG-lipids) [15, 16]	Block copolymers composed of PEG and cationic polyamino acid [3-5]
**Structure**	A core-sell structure with mRNA encapsulated in the core in general but highly variable in structure [19-20]	A core-shell structure surrounded by dense PEG palisade with mRNA encapsulated in the core [3-5]
**Assembling**	Self-assembled mainly driven by hydrophobic interactions and guided by electrostatic interactions [18]	Self-assembled by electrostatic interactions [3-5]
**Size**	Changeable based on applications and components [18, 21]	Regulated between 50-100 nm [6]
**Administration route**	Applicable for various routes e.g., intravenous (i.v.) and local administration.	Various Local administration routes [9-15] and hydrodynamic intravenous injection [8]
**Biodistribution after i.v. administration**	Accumulations in the liver and lymph nodes [22, 23]	-
**Immune response after administration**	Considerable immune responses, which could work as adjuvant for vaccination [1, 2]	Less or no immune response [6, 7, 9, 14]

References for Table S1

1. Kedmi, R.; Ben-Arie, N.; Peer, D. The systemic toxicity of positively charged lipid nanoparticles and the role of Toll-like receptor 4 in immune activation. *Biomaterials* **2010**, *31*, 6867-6875, doi:10.1016/j.biomaterials.2010.05.027.
2. Sedic, M.; Senn, J.J.; Lynn, A.; Laska, M.; Smith, M.; Platz, S.J.; Bolen, J.; Hoge, S.; Bulychev, A.; Jacquinet, E.; et al. Safety Evaluation of Lipid Nanoparticle-Formulated Modified mRNA in the Sprague-Dawley Rat and Cynomolgus Monkey. *Vet Pathol* **2018**, *55*, 341-354, doi:10.1177/0300985817738095.
3. Kanayama, N.; Fukushima, S.; Nishiyama, N.; Itaka, K.; Jang, W.D.; Miyata, K.; Yamasaki, Y.; Chung, U.I.; Kataoka, K. A PEG-based biocompatible block catiomer with high buffering capacity for the construction of polyplex micelles showing efficient gene transfer toward primary cells. *ChemMedChem* **2006**, *1*, 439-444, doi:10.1002/cmdc.200600008.
4. Uchida, H.; Miyata, K.; Oba, M.; Ishii, T.; Suma, T.; Itaka, K.; Nishiyama, N.; Kataoka, K. Odd-even effect of repeating aminoethylene units in the side chain of N-substituted

polyaspartamides on gene transfection profiles. *J Am Chem Soc* **2011**, *133*, 15524-15532, doi:10.1021/ja204466y.
5. Uchida, H.; Itaka, K.; Nomoto, T.; Ishii, T.; Suma, T.; Ikegami, M.; Miyata, K.; Oba, M.; Nishiyama, N.; Kataoka, K. Modulated protonation of side chain aminoethylene repeats in N-substituted polyaspartamides promotes mRNA transfection. *J Am Chem Soc* **2014**, *136*, 12396-12405, doi:10.1021/ja506194z.
6. Itaka, K.; Kataoka, K. Progress and prospects of polyplex nanomicelles for plasmid DNA delivery. *Curr Gene Ther* **2011**, *11*, 457-465, doi:10.2174/156652311798192879.
7. Uchida, S.; Itaka, K.; Uchida, H.; Hayakawa, K.; Ogata, T.; Ishii, T.; Fukushima, S.; Osada, K.; Kataoka, K. In vivo messenger RNA introduction into the central nervous system using polyplex nanomicelle. *PLoS One* **2013**, *8*, e56220, doi:10.1371/journal.pone.0056220.
8. Matsui, A.; Uchida, S.; Ishii, T.; Itaka, K.; Kataoka, K. Messenger RNA-based therapeutics for the treatment of apoptosis-associated diseases. *Sci Rep* **2015**, *5*, 15810, doi:10.1038/srep15810.
9. Aini, H.; Itaka, K.; Fujisawa, A.; Uchida, H.; Uchida, S.; Fukushima, S.; Kataoka, K.; Saito, T.; Chung, U.I.; Ohba, S. Messenger RNA delivery of a cartilage-anabolic transcription factor as a disease-modifying strategy for osteoarthritis treatment. *Sci Rep* **2016**, *6*, 18743, doi:10.1038/srep18743.
10. Lin, C.Y.; Perche, F.; Ikegami, M.; Uchida, S.; Kataoka, K.; Itaka, K. Messenger RNA-based therapeutics for brain diseases: An animal study for augmenting clearance of beta-amyloid by intracerebral administration of neprilysin mRNA loaded in polyplex nanomicelles. *J Control Release* **2016**, *235*, 268-275, doi:10.1016/j.jconrel.2016.06.001.
11. Crowley, S.T.; Fukushima, Y.; Uchida, S.; Kataoka, K.; Itaka, K. Enhancement of Motor Function Recovery after Spinal Cord Injury in Mice by Delivery of Brain-Derived Neurotrophic Factor mRNA. *Mol Ther Nucleic Acids* **2019**, *17*, 465-476, doi:10.1016/j.omtn.2019.06.016.
12. Lin, C.Y.; Crowley, S.T.; Uchida, S.; Komaki, Y.; Kataoka, K.; Itaka, K. Treatment of Intervertebral Disk Disease by the Administration of mRNA Encoding a Cartilage-Anabolic Transcription Factor. *Mol Ther Nucleic Acids* **2019**, *16*, 162-171, doi:10.1016/j.omtn.2019.02.012.
13. Fukushima, Y.; Uchida, S.; Imai, H.; Nakatomi, H.; Kataoka, K.; Saito, N.; Itaka, K. Treatment of ischemic neuronal death by introducing brain-derived neurotrophic factor mRNA using polyplex nanomicelle. *Biomaterials* **2021**, *270*, 120681, doi:10.1016/j.biomaterials.2021.120681.
14. Oyama, N.; Kawaguchi, M.; Itaka, K.; Kawakami, S. Efficient Messenger RNA Delivery to the Kidney Using Renal Pelvis Injection in Mice. *Pharmaceutics* **2021**, *13*, doi:10.3390/pharmaceutics13111810.
15. Hashimoto, Y.; Kuniishi, H.; Sakai, K.; Fukushima, Y.; Du, X.; Yamashiro, K.; Hori, K.; Imamura, M.; Hoshino, M.; Yamada, M.; et al. Brain Dp140 alters glutamatergic transmission and social behaviour in the mdx52 mouse model of Duchenne muscular dystrophy. *Prog Neurobiol* **2022**, *216*, 102288, doi:10.1016/j.pneurobio.2022.102288.
16. Blanco, E.; Shen, H.; Ferrari, M. Principles of nanoparticle design for overcoming biological barriers to drug delivery. *Nat Biotechnol* **2015**, *33*, 941-951, doi:10.1038/nbt.3330.
17. Semple, S.C.; Akinc, A.; Chen, J.; Sandhu, A.P.; Mui, B.L.; Cho, C.K.; Sah, D.W.; Stebbing, D.; Crosley, E.J.; Yaworski, E.; et al. Rational design of cationic lipids for siRNA delivery. *Nat Biotechnol* **2010**, *28*, 172-176, doi:10.1038/nbt.1602.
18. Kim, J.; Eygeris, Y.; Gupta, M.; Sahay, G. Self-assembled mRNA vaccines. *Adv Drug Deliv Rev* **2021**, *170*, 83-112, doi:10.1016/j.addr.2020.12.014.
19. Yanez Arteta, M.; Kjellman, T.; Bartesaghi, S.; Wallin, S.; Wu, X.; Kvist, A.J.; Dabkowska, A.; Székely, N.; Radulescu, A.; Bergenholtz, J.; et al. Successful reprogramming of cellular protein production through mRNA delivered by functionalized lipid nanoparticles. *Proc Natl Acad Sci U S A* **2018**, *115*, E3351-e3360, doi:10.1073/pnas.1720542115.

20. Kulkarni, J.A.; Witzigmann, D.; Leung, J.; van der Meel, R.; Zaifman, J.; Darjuan, M.M.; Grisch-Chan, H.M.; Thöny, B.; Tam, Y.Y.C.; Cullis, P.R. Fusion-dependent formation of lipid nanoparticles containing macromolecular payloads. *Nanoscale* **2019**, *11*, 9023-9031, doi:10.1039/c9nr02004g.
21. Heyes, J.; Hall, K.; Tailor, V.; Lenz, R.; MacLachlan, I. Synthesis and characterization of novel poly(ethylene glycol)-lipid conjugates suitable for use in drug delivery. *J Control Release* **2006**, *112*, 280-290, doi:10.1016/j.jconrel.2006.02.012.
22. Hou, X.; Zaks, T.; Langer, R.; Dong, Y. Lipid nanoparticles for mRNA delivery. *Nat Rev Mater* **2021**, *6*, 1078-1094, doi:10.1038/s41578-021-00358-0.
23. Pardi, N.; Tuyishime, S.; Muramatsu, H.; Kariko, K.; Mui, B.L.; Tam, Y.K.; Madden, T.D.; Hope, M.J.; Weissman, D. Expression kinetics of nucleoside-modified mRNA delivered in lipid nanoparticles to mice by various routes. *J Control Release* **2015**, *217*, 345-351, doi:10.1016/j.jconrel.2015.08.007.
